# Prostate Cancer in the MENA Region: Attributable Burden of Behavioral and Environmental Exposures

**DOI:** 10.3390/toxics14010096

**Published:** 2026-01-21

**Authors:** Magie Tamraz, Razan Al Tartir, Sara El Meski, Sally Temraz

**Affiliations:** 1 Department of Epidemiology and Population Health, Faculty of Health Sciences, American University of Beirut, Riad El Solh, Beirut 1107, Lebanon; mnt04@mail.aub.edu; 2 Division of Oncology/Hematology, Department of Internal Medicine, American University of Beirut Medical Center, Riad El Solh, Beirut 1107, Lebanon; ra464@aub.edu.lb (R.A.T.); se153@aub.edu.lb (S.E.M.)

**Keywords:** prostate cancer, Middle East and North Africa, population attributable fraction, nitrate, water quality, tobacco smoking, physical inactivity, environmental exposure

## Abstract

Background: Prostate cancer in the Middle East and North Africa (MENA) region is shaped by a complex interplay of behavioral and environmental risk factors, yet comprehensive estimates of preventable cases remain scarce. To address this gap, we estimated population-attributable fractions (PAFs) for a range of modifiable exposures among men aged 50 years and older and assessed potential reductions in incidence under feasible intervention scenarios. Methods: Regional prevalence data were combined with relative risks from meta-analyses to compute closed-form PAFs for tobacco smoking, obesity, physical inactivity, high dairy and calcium intake, heavy alcohol use, drinking water nitrates, trihalomethanes, arsenic, lead, selenium status, ambient PM_2.5_ and NO_2_, and occupational diesel exhaust, covering an estimated 47 million men. Estimates were validated using a synthetic cohort simulation of 100,000 individuals, with uncertainty quantified through Monte Carlo sampling. Results: Results showed that drinking water nitrate exposure accounted for the largest single fraction (17.4%), followed by tobacco smoking (9.5%), physical inactivity (6.7%), and trihalomethane exposure (5.0%), while other exposures contributed smaller but meaningful shares. Joint elimination of all exposures projected a 45.5% reduction in incidence, and simultaneous feasible reductions in four targeted exposures yielded a combined potential impact fraction of 12.1%. Conclusions: These findings suggest that integrated water quality management, tobacco control, lifestyle interventions, and targeted environmental surveillance should be prioritized to reduce prostate cancer burden in the MENA region. However, estimates of drinking-water nitrate exposure rely on limited evidence from a single case–control study with a relatively small sample size, and should therefore be considered exploratory and primarily hypothesis-generating.

## 1. Introduction

Prostate cancer remains a global burden afflicting around 1.5 million males globally, corresponding to an age-standardized incidence rate of 30.7 per 100,000 men [1]. Despite knowledge about the etiology of the disease and substantial improvement in treatment modalities, prostate cancer mortality remains high, with over 300,000 deaths annually, with an age-standardized mortality rate of 7.7 per 100,000 men [1].

In the Middle East and North African (MENA) region, the age-standardized rates of prostate incidence and death in 2019 were 23.7 and 11.7 per 100,000 population, respectively [2]. The higher mortality rate in the region could be directly attributed to advanced stage at diagnosis, when symptoms of the disease dominate, with 35% of men presenting with clinical T3 or T4 disease and 54% with Stage IV disease [3]. Several factors, most notably limited public awareness and inadequate prostate-specific antigen (PSA) screening, could be attributed to the large proportion of men being diagnosed with prostate cancer at an advanced or metastatic stage. In the MENA region, factors that have been implicated in driving the region’s rising incidence of the disease include modifiable and non-modifiable risk factors. Advancing age, familial predisposition, and inherited genetic susceptibility constitute the main non-modifiable determinants of prostate cancer risk. Modifiable factors, on the other hand, include tobacco smoking, obesity and central adiposity, Westernized high-fat dietary patterns, physical inactivity, and exposure to environmental carcinogens including waterborne and airborne pollutants [4,5].

Previous global and regional studies have estimated that behavioral and environmental exposures account for a meaningful share of prostate cancer burden. For example, analyses from the Global Burden of Disease (GBD) project reported that high body mass index and tobacco use together explained approximately 15–20% of prostate cancer disability adjusted life years worldwide [6]. Occupational studies in the UK further suggested that workplace exposures such as diesel exhaust and pesticides contribute around 2% of prostate cancer cases [7]. More broadly, umbrella reviews of prospective studies have highlighted consistent associations between obesity, smoking, and dietary patterns with increased prostate cancer risk [8]. At the same time, genetic susceptibility and advancing age remain dominant non-modifiable determinants of disease risk [8]. These findings underscore that while modifiable exposures explain a non-trivial fraction of prostate cancer burden, they coexist with strong inherited and biological contributors not captured in population attributable fraction analyses.

By stratifying guidance according to resource availability, a recent study provided consensus-based recommendations on optimal diagnostic imaging and stage-specific treatment for managing high-risk and advanced prostate cancer across the MENA region [9]. Building on these consensus-based recommendations, our study will quantify the public health impact of key behavioral risk factors and waterborne pollutants by estimating their respective population-attributable fractions (PAFs) in the target MENA population. Calculating the PAF for individual risk factors is crucial because it translates relative risks into the proportion of prostate cancer cases that could be prevented if a given exposure were eliminated or reduced. To date, no published study has performed this analysis specifically for prostate cancer in the MENA region. By quantifying each factor’s share of the overall disease burden, whether for smoking, obesity, dietary components, physical inactivity, or waterborne and airborne contaminants, we can prioritize public health interventions, tailor screening and education campaigns to the highest-impact exposures, and model the potential reduction in incidence and mortality that would follow targeted prevention strategies.

## 2. Materials and Methods

### 2.1. Study Design

In this retrospective data analysis, the exposure to the risk is examined in relation to prostate cancer occurrence. The PAF was used to estimate the role of modifiable risk factors on prostate cancer incidence in the MENA region. To quantify the contribution of a risk factor to cancer, two main components are needed; the proportion of the population subjected to the risk and the relative risk of cancer associated with that risk factor. The proportion exposed to the risk is quantified by obtaining the prevalence of each risk factor and multiplying it by the cohort under study.

### 2.2. Prevalence of Risk Factors

In order to study the impact of behavioral risk factors and environmental exposures on the risk of prostate cancer in the MENA region, we estimated the exposed male population aged 50 years and older in the MENA region at roughly 47 million men based on regional prevalence data by the Global Burden of Disease Study 2019 [10]. To keep our approach transparent and comparable across risk factors, we assumed a uniform age-group exposure pattern, meaning that whatever fraction of the general adult population is exposed, the same fraction applies to our ≥50 years male cohort.

The prevalence data for tobacco use, obesity (BMI ≥ 30 kg/m^2^), and physical inactivity in males ≥50 years were 25%, 30%, and 40% obtained from a report by the World Health Organization (WHO) on Noncommunicable Disease Country Profiles in 2021 [11]. Prevalence estimates for dairy and calcium intake were modeled from the Food and Agriculture Organization (FAO) Food Balance Sheets 2015–2020, yielding an approximate value of 35% of men ≥50 years in MENA exceeding 1000 mg/day [12]. These figures reflect food supply availability rather than actual individual intake, and therefore likely overestimate true consumption. As for heavy alcohol consumption, data were obtained from the WHO Global status report on alcohol and health, which found that heavy alcohol consumption patterns are reported by around 5% of this demographic [13]. This estimate reflects current heavy drinking prevalence, defined by WHO as consumption of ≥60 g of pure alcohol on at least one occasion in the past 30 days, rather than lifetime exposure.

For drinking-water nitrates, from the FAO AQUASTAT database, about 40% of the MENA population depends on untreated groundwater for their daily supply [14]. Meanwhile, global water-quality surveys show that roughly three-quarters of these groundwater sources exceed the WHO’s 50 mg/L nitrate threshold [15]. Weighting those figures yields our 30% nitrate-exposure estimate. Trihalomethane (THM) exposure is driven by municipal chlorination practices. The WHO/UNICEF Joint Monitoring Program reports that around 70% of MENA residents use centralized water networks [16], while the multi-pathway public drinking-water assessment by Semerjian et al. revealed that about half of those supplies have THM concentrations above 49 µg/L [17]. Multiplying coverage by exceedance gives our 35% THM-exposure estimate. National water-quality audits and WHO reports indicate that roughly 35 percent of MENA households draw directly from wells, and about half of those wells exceed WHO limits for arsenic (10 µg/L) or lead (10 µg/L) [14,15]. Together, these underpin our 18 percent estimate for arsenic/lead exposures. For exposures showing apparent protective associations (e.g., selenium, calcium, protein), deficiency prevalence data were not consistently available in regional datasets. Therefore, these estimates should not be interpreted as evidence supporting supplementation, but rather as epidemiological associations requiring cautious interpretation.

As for airborne exposures, ambient PM_2.5_ and NO_2_ exposure rates were taken from the WHO Ambient Air Quality Database [18]. Urbanization rates in MENA are around 60%, and the WHO’s Ambient Air Pollution database shows that PM_2.5_ and NO_2_ levels in almost every major city there exceed guideline values [19]. Finally, occupational exposure to diesel exhaust was estimated at about 2% among adult men based on International Labor Organization employment-sector data and industry-specific surveys showing that roughly one in fifty male workers in MENA routinely handles or works near heavy diesel engines [20,21].

For clarity, we note that some prevalence estimates were derived from MENA-specific surveillance sources (e.g., WHO Noncommunicable Disease Country Profiles for tobacco, obesity, and physical inactivity; FAO AQUASTAT and WHO/UNICEF JMP for water quality; WHO Ambient Air Quality Database for MENA cities), while others relied on global datasets applied to MENA populations (e.g., FAO Food Balance Sheets for dairy/calcium intake, WHO Global Status Report on Alcohol for heavy drinking, selenium status meta-analyses, and ILO sectoral data for occupational diesel exposure). This distinction is important for interpreting comparability across exposures.

### 2.3. Relative Risk for Each Risk Factor

Because no meta-analysis specific to the MENA region is available, we relied on a comprehensive umbrella review of global observational studies and meta-analyses to derive relative risk (RR) estimates. Table 1 shows the relative risks associated with each behavioral risk factor and the sources the data were obtained from. Also, the estimated exposed population (calculated by multiplying the prevalence by 47 million) to each behavioral risk factor is shown. Table 2 presents the relative risks associated with each environmental exposure and their sources. For nitrate exposure, the relative risk estimate was derived from a single case–control study conducted in Spain (97 cases and 927 controls). In contrast to other exposures for which pooled meta-analyses were available, this limited evidence base should be considered when interpreting the robustness and comparability of the PAF estimates. Table 2 also shows the estimated exposed population to each environmental pollutant.

### 2.4. Statistical Methods

#### 2.4.1. PAF Calculation

To estimate the contribution of a risk factor to disease burden, it is expressed as the percentage of disease that is caused by a specific risk factor. The attributable risk in a population depends on the prevalence of the risk factor and the strength of its association (relative risk) with the disease.

The PAF for each risk factor was computed using the formula:

PAF = Pe × (RR − 1)/[Pe × (RR − 1) + 1],

where Pe represents the prevalence of exposure in the population, and RR is the relative risk of CRC associated with each contaminant.

It should be noted that PAF calculations assume a causal relationship between exposure and disease. For exposures where the evidence base is primarily observational (e.g., THMs, PM_2.5_, NO_2_, diesel exhaust), these estimates should be interpreted with caution and considered indicative rather than definitive.

#### 2.4.2. Synthetic Cohort Simulation

We generated a virtual cohort of 100,000 men aged 40–80 years to validate our prevalence-based PAF estimates. For each risk factor, including both behavioral exposures (e.g., smoking, obesity, physical inactivity, dairy and calcium intake, heavy alcohol) and environmental exposures (e.g., nitrates, THMs, arsenic, lead, selenium, PM_2.5_, NO_2_, diesel exhaust), we created a binary indicator drawn from a Bernoulli distribution with probability equal to the reported prevalence in MENA men ≥ 50 years. We then calibrated a baseline log-odds intercept via numerical root-finding so that, when summed with each exposure’s log-relative risk, the average simulated prostate cancer incidence matched the region’s age-standardized rate. Each man’s disease log-odds was calculated as this calibrated intercept plus the sum of his exposure indicators multiplied by the log of their RRs. We converted log-odds to a probability with the logistic function and drew case status from a Bernoulli trial. To estimate cohort-based PAFs, we repeated the simulation for each factor set to zero (while holding all others at their observed prevalence) and measured the proportional reduction in incidence, directly comparing these cohort-derived fractions to the closed-form PAFs.

#### 2.4.3. Sensitivity Analysis

We assessed uncertainty around our PAFs using two approaches. First, we performed a probabilistic (Monte Carlo) analysis with 1000 iterations. In each iteration, every RR was sampled from a log-normal distribution calibrated to its published 95% CI on the log scale, and every prevalence was drawn from a beta distribution fitted to its survey-derived confidence limits. We reran the full cohort simulation for each draw and recorded the resulting PAFs, summarizing their means and 2.5th–97.5th percentiles as our 95% uncertainty intervals. Second, we conducted one-way sensitivity analyses in which each RR and each prevalence parameter was individually varied across its confidence bounds while holding all other inputs at their point estimates.

To support the development of the synthetic cohort simulation and sensitivity analyses, we employed Microsoft Copilot (version 1.25064.139.0, accessed via Windows 11) to generate the initial R code framework and logical structure. Copilot was used specifically to accelerate the coding of simulation routines and probabilistic sampling functions. All scripts were subsequently refined, implemented, and executed in R (https://posit.cloud, version 4.3.3 (29 February 2024)), where we performed output verification, parameter calibration, and sensitivity analyses to ensure the robustness and reproducibility of the simulation outcomes.

#### 2.4.4. Joint PAF

We calculated the combined burden of all risk factors by avoiding simple summation. Let PAF_1_, PAF_2_, …, PAF_k_ be the individual fractions for k exposures. The joint fraction was as follows:

Joint PAF = 1 − [(1 − PAF_1_) × (1 − PAF_2_) × … × (1 − PAF_k_)]

#### 2.4.5. Potential Impact Fraction

To translate our all-or-nothing PAFs into achievable public-health targets, we estimated Potential Impact Fractions (PIFs) for four high-impact risk factors: tobacco smoking, physical inactivity, nitrate exposure, and high calcium intake. For each factor, we obtained its current (baseline) prevalence in MENA men aged ≥50 years from survey data and defined a realistic target prevalence equal to baseline minus 10 percentage points. We then applied the standard PIF formula:

PIF = [p_0_·(RR − 1) − p_1_·(RR − 1)]/[p_0_·(RR − 1) + 1]

where p_0_ is the baseline prevalence; p_1_ is the target prevalence; and RR is the relative risk from our primary analysis.

To estimate the joint impact of simultaneous reductions, we assumed independent effects across factors and combined individual PIFs multiplicatively:

Combined PIF = 1 − ∏_i_(1 − PIF_i_).

All calculations were conducted in R (https://posit.cloud, version 4.3.3 (29 February 2024)) using built-in vector operations.

## 3. Results

Using the RR for each behavioral and environmental risk factor and the population exposed, we calculate the PAF values for each risk factor as outlined in Table 3. Among the behavioral risk factors examined, cigarette smoking, physical inactivity, and high calcium intake accounted for the largest proportions of prostate cancer cases, with attributable fractions of 9.5%, 6.7%, and 5.6%. Among environmental contaminants, elevated nitrate exposure in drinking water posed the greatest risk, accounting for 17.4% of cases, followed by THM exposure with a PAF of 5.0%. It is important to note that the nitrate-attributable PAF was based on a single case–control study conducted in Spain, characterized by a relatively small sample size. As such, the strength of the underlying evidence is limited, and the estimate should be interpreted as exploratory and hypothesis-generating rather than definitive.

### 3.1. Cohort-Based PAF Estimates

Cohort-based PAFs derived from the calibrated simulation closely matched the closed-form, prevalence-based values. Among behavioral risk factors, tobacco smoking accounted for the largest fraction of preventable prostate cancer cases (9.5%), followed by physical inactivity (6.7%) and high calcium intake (5.6%). Obesity, high dairy intake, and heavy alcohol consumption contributed smaller PAFs (3.5%, 3.0%, and 0.9%, respectively). Among environmental exposures, nitrate contamination in drinking water had the greatest impact (17.4%), followed by THMs (5.0%), arsenic (3.1%), PM_2.5_ (3.5%), and NO_2_ (2.4%). Selenium intake exhibited a protective effect (PAF = –4.1%), and occupational diesel exposure accounted for 0.5% of cases. Table 4 displays these cohort-based PAF estimates.

### 3.2. Sensitivity Analysis

Probabilistic Monte Carlo sensitivity analysis (n = 1000) generated 95% uncertainty intervals around each PAF (mean ± 95% UI), and one-way sensitivity ranges were also calculated (see Supplementary Tables S1 and S2). Both analyses confirm the robustness of our key findings: PAFs for tobacco, physical inactivity, high calcium intake, and nitrate exposure remain substantially elevated even under plausible input variability.

### 3.3. Joint PAF and PIF

**Figure 1:**
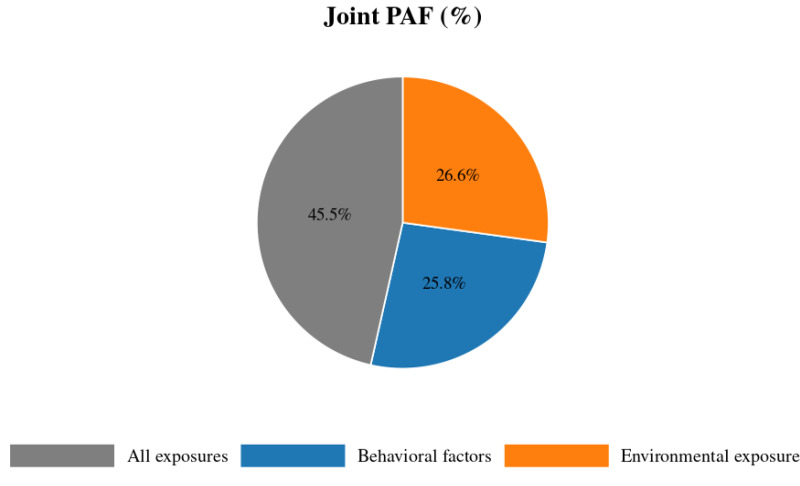
Joint PAFs for behavioral factors alone, environmental factors (including the protective effect of selenium), and for all factors together. Exposure categories are overlapping: individuals may contribute to both behavioral and environmental groups. The “all factors together” category represents the combined burden across domains, calculated using the joint PAF formula. Note: Categories are non-exclusive; percentages do not sum to 100%.

Eliminating all six behavioral risks at once could potentially avert 25.8% of prostate cancer cases, compared with the sum of individual PAFs (29.2%), reflecting overlap among exposures. While removing all environmental exposures simultaneously yields a 26.6% reduction in cases, slightly diminished by the protective selenium effect (PAF = −4.1%). Eradicating every measured exposure, both behavioral and environmental, would translate into a 45.5% drop in prostate cancer incidence, underscoring the potential impact of comprehensive preventive strategies targeting multiple risk pathways (Figure 1).

**Figure 2:**
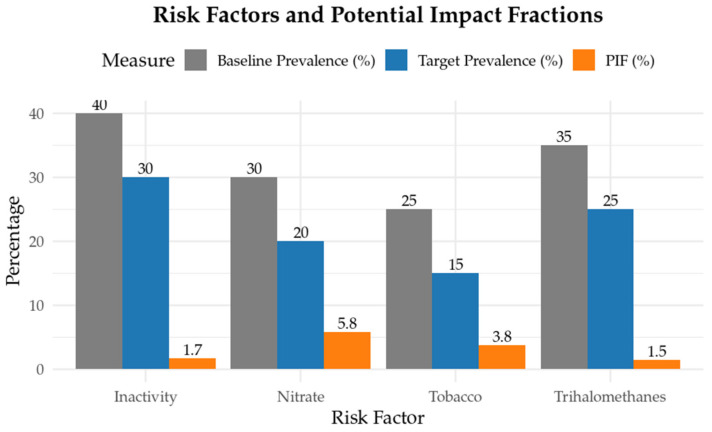
Potential impact fractions for selected risk factors.

Figure 2 presents the estimated reduction in prostate cancer burden associated with a 10–percentage-point absolute decrease in the prevalence of THMS, physical inactivity, nitrate exposure, and tobacco smoking. Because calcium intake estimates derived from FAO supply data likely overstate actual consumption in MENA populations, we elected to substitute THMs as the next highest contributor after calcium (PAF = 5.0% vs. 5.6% for calcium). THMs were selected based on their robust epidemiological evidence (RR = 1.15, 95% CI: 0.95–1.40) and substantial prevalence (35%, ~16.5 million men), ensuring that the modeled burden estimates remained both realistic and evidence-based.

A realistic 10 percentage-point reduction in these four risk factors corresponds to PIFS of 1.5%, 1.7%, 5.8%, and 3.8%, respectively. When these four achievable targets are met simultaneously, the combined potential impact fraction reaches 12.1%, demonstrating that modest improvements across multiple exposures can collectively avert more than one-tenth of prostate cancer cases.

## 4. Discussion

This analysis provides a regionally focused, quantitative estimate of the fraction of prostate cancer cases among men aged 50 years and older in the MENA region that can be attributed to a comprehensive set of modifiable behavioral and environmental exposures. By combining prevalence-based PAF calculations with cohort-based simulation and probabilistic uncertainty analysis, we produced robust point estimates and uncertainty bounds to inform regional public-health prioritization. The results identify nitrate contamination of drinking water, tobacco smoking, physical inactivity, and high calcium intake as the principal contributors to prostate cancer burden in the region, with additional but smaller contributions from disinfection by-products (THMs), arsenic and lead, ambient air pollution, and occupational diesel exhaust.

The elevated PAF for nitrate in drinking water (PAF = 17.4%) likely reflects the combination of widespread reliance on untreated or minimally treated groundwater in parts of MENA and the biologic plausibility that ingested nitrate can be reduced to nitrite and participate in endogenous formation of N-nitroso compounds, which have genotoxic potential. This observation underscores the importance of water-quality interventions such as groundwater monitoring, targeted point-of-use treatment, and agricultural nutrient management to reduce human nitrate exposure [2,29]. THMs, formed during chlorination of organic-rich waters, were also associated with a measurable burden (PAF = 5.0%). This is consistent with recent systematic reviews and dose–response meta-analyses linking long-term THM exposure to elevated cancer risk and mechanistic evidence of genotoxicity and oxidative stress from specific THM species [35].

Tobacco smoking accounted for a significant proportion of preventable prostate cancer cases (PAF = 9.5%), driven both by a large exposed population and elevated RRs for advanced disease, as documented in pooled analyses. Accordingly, strengthening tobacco control policies, expanding cessation services, and implementing targeted screening initiatives for middle-aged and older men should be prioritized across the region, particularly given the variation in cigarette and waterpipe use across countries [5,22]. Physical inactivity (PAF = 6.7%) and obesity (PAF = 3.5%) represent a lifestyle-related risk cluster. These factors are linked to prostate tumorigenesis and progression through biologically plausible mechanisms, including disruption of the insulin–insulin growth factor (IGF) axis, alterations in sex hormone levels, chronic low-grade inflammation, and adipokine-mediated signaling. Regional analyses report increasing levels of sedentary behavior and overweight in MENA, reinforcing the need for interventions that promote long-term physical activity and healthy weight management [4,10].

Dietary findings require precise interpretation. The prevalence estimates for high calcium intake used in our modeling were derived from FAO Food Balance Sheets, which reflect food supply rather than actual consumption. As such, the calculated PAF of 5.6% may overstate the true burden attributable to calcium, given that national dietary surveys in MENA consistently report mean intakes well below 1000 mg/day [36,37,38]. This uncertainty underscores the need for future epidemiological studies to quantify the actual proportion of men exceeding the ≥1000 mg/day threshold, thereby providing more accurate prevalence data to inform risk estimates and dietary guidance.

Environmental exposures contributed meaningfully to prostate cancer burden in the MENA region. Chronic arsenic exposure (PAF = 3.1%) is supported by strong biological evidence, including mechanisms involving oxidative stress, DNA damage, and epigenetic changes. Lead (PAF = 0.7%) may be relevant in localized areas with legacy industrial pollution or aging water infrastructure, where contamination persists despite regulatory efforts [31,39]. Air pollution also played a role: ambient PM_2.5_ (PAF = 3.5%) and NO_2_ (PAF = 2.3%) were associated with prostate cancer burden, consistent with recent meta-analyses linking long-term exposure to combustion-related pollutants with systemic inflammation and genotoxic effects [33]. Occupational exposure to diesel engine exhaust accounted for a smaller share (≈0.5%), but epidemiological studies suggest increased risk of aggressive prostate cancer among workers with high exposure, particularly in transport and industrial sectors [34].

Selenium intake showed a protective association (PAF = −4.1%), consistent with evidence of its role in antioxidant defense through selenoproteins. However, findings from randomized trials are mixed, and benefits appear to depend on baseline selenium status and genetic factors. As such, public health strategies should focus on ensuring adequate dietary intake rather than promoting high-dose supplementation [40]. It is important to emphasize that protective associations observed in our analysis should not be interpreted as evidence supporting supplementation. Rather, they highlight the potential role of deficiency states, which were not directly modeled due to data limitations. Supplementation should not be considered a preventive strategy for prostate cancer based on our estimates.

Joint exposure modeling highlights the potential impact of integrated prevention strategies. Eliminating the behavioral risk factors assessed could prevent an estimated 25.8% of prostate cancer cases, while addressing environmental exposures could reduce incidence by 26.6%. Tackling both domains together could avert nearly half of all cases under PAF assumptions. Moreover, the PIF analysis suggests that modest, achievable reductions, such as 10 percentage-point decreases in smoking, physical inactivity, nitrate exposure, and THMs, could collectively prevent approximately 12.1% of cases. These findings offer practical targets for public health interventions and policy planning.

This study has several strengths. It integrates behavioral and environmental risk factors into a single regional PAF framework, uses closed-form PAFs validated through cohort-based simulation, and applies probabilistic sensitivity analysis to account for uncertainty in input parameters. These methods enhance the robustness and policy relevance of the findings and draw attention to exposures, particularly waterborne contaminants, that are often underrepresented in cancer burden assessments focused primarily on lifestyle factors.

Nonetheless, several limitations should be noted. Exposure prevalence estimates were drawn from regional and international surveillance sources and applied uniformly to men aged 50 and older, which may obscure subnational variation in exposure levels and water source reliance. This assumption of uniform prevalence across such a large and heterogeneous population is unlikely to hold, and exposure variability by geography, socioeconomic status, and occupation may introduce bias into the PAF estimates. Although many prevalence estimates were MENA-specific, some (e.g., dairy/calcium intake, alcohol consumption, selenium status) relied on global datasets applied to MENA populations. This may introduce uncertainty and affect comparability across exposures, underscoring the need for more region-specific surveillance data. For calcium intake, prevalence estimates were modeled from FAO Food Balance Sheets, which reflect supply rather than actual consumption. This may overestimate the proportion of men exceeding 1000 mg/day. Relative risks were obtained from global meta-analyses due to the limited availability of high-quality prospective cohort data from MENA, which could affect the accuracy of PAF estimates if regional differences in risk exist. In particular, the relative risk estimate for drinking water nitrate exposure was based on a single case–control study (Spain, 97 cases and 927 controls) rather than pooled analyses. This limited evidence base may reduce robustness and comparability, and the conclusion that nitrate accounts for the largest single attributable fraction should be interpreted with caution until further meta-analytic evidence becomes available. Furthermore, the regional prevalence estimates of heavy alcohol consumption (5%) reflect current drinking patterns and may mask heterogeneity across MENA countries, where cultural and religious contexts strongly influence alcohol use. Some environmental exposures were modeled as binary variables exceeding regulatory thresholds rather than using continuous exposure–response relationships. PAF calculations assume causal associations and, for single-factor estimates, independence from unmeasured confounding. While joint PAFs account for measured overlap, residual confounding and interactions may still influence results. Importantly, the calculation of joint PAFs relies on the assumption of independence be-tween exposures. However, several of the environmental factors considered in this study, such as PM_2.5_, NO_2_, and urbanization, are likely to be correlated. This interdependence may result in an overestimation of the combined preventable risk. Future analyses should incorporate multivariable approaches that account for exposure covariance to provide more conservative and accurate estimates. Finally, because PAFs assume causality, our prevention potential estimates for exposures supported mainly by observational evidence (such as THMs, PM_2.5_, NO_2_, and diesel exhaust) should be interpreted cautiously. While these associations are biologically plausible and supported by epidemiological data, further mechanistic and prospective studies are required to strengthen causal inference.

## 5. Conclusions

A significant proportion of prostate cancer cases among men aged 50 and older in the MENA region is linked to modifiable behavioral and environmental exposures. Notably, nitrate contamination in drinking water, tobacco use, and physical inactivity stand out as key contributors. This highlights the need for coordinated efforts to improve water quality, strengthen tobacco control, and promote active lifestyles, supported by enhanced environmental monitoring and targeted remediation.

To refine and tailor these estimates, future priorities should include regionally representative cohort studies with standardized exposure assessments, spatially detailed tracking of water and air pollutants, and practical intervention trials. Examples include point-of-use water treatment, improved agricultural nutrient practices, culturally adapted tobacco cessation programs, and community-based physical activity initiatives. Together, these policies, surveillance, and research efforts can help translate the estimated attributable fractions into actionable, evidence-based public health strategies.

## Figures and Tables

**Table 1:** Population at risk and relative risk for behavioral risk factors associated with prostate cancer in the MENA region.

Behavioral Risk Factor	Relative Risk (95% CI)	Reference	Prevalence in MENA Men ≥ 50 Years	Estimated Exposed Population (Millions)
Tobacco smoking (ever vs. never)	1.42 (1.20–1.68)	[22]	25% of men smoke	11.75
Obesity (BMI ≥ 30 vs. <25 kg/m^2^)	1.12 (1.06–1.19)	[23]	30% of men are obese	14.10
Physical inactivity (lowest vs. highest activity)	1.18 (1.08–1.29) inverse of protective RR 0.85 (0.78–0.94)	[24]	40% of men insufficiently active	18.80
High dairy product intake (highest vs. lowest)	1.09 (1.02–1.16)	[25]	35% of men consume high amounts of dairy	16.45
High calcium intake (≥1000 mg/day vs. <500 mg/day)	1.17 (1.06–1.28)	[26]	35% of men exceed 1000 mg/day	16.45
Heavy alcohol consumption (high vs. none)	1.18 (1.10–1.26)	[27]	5% of men are heavy drinkers	2.35

**Table 2:** Population at risk and relative risk for environmental risk factors associated with prostate cancer in the MENA region.

Contaminant	Relative Risk (95% CI)	Reference	Exposure Prevalence (% Men ≥ 50)	Estimated Exposed Men ≥ 50
Nitrate (drinking water)	1.70 (1.20–2.41)	[28]	30%	14.1 million
THMs	1.15 (0.95–1.40)	[29]	35%	16.5 million
Arsenic	1.18 (1.06–1.30)	[30]	18%	8.5 million
Lead	1.04 (1.02–1.05) per study-specific increment	[31]	18%	8.5 million
Selenium (protective)	0.86 (0.78–0.94) per unit increase	[32]	28%	13.2 million
PM_2.5_ (ambient air)	1.06 (1.02–1.10) per 5 µg/m^3^ increase	[33]	60%	28.2 million
NO_2_ (ambient air)	1.04 (1.01–1.07) per 10 µg/m^3^ increase	[33]	60%	28.2 million
Diesel exhaust (occup.)	RR 1.24 (0.96–1.61) ^†^ RR 1.27 (0.80–2.01) ^‡^	[34]	2%	0.94 million

^†^ Change from 25th to 75th percentile of cumulative diesel-engine exhaust exposure. ^‡^ Change from 75th to 95th percentile of cumulative diesel-engine exhaust exposure.

**Table 3:** Population-attributable fractions (PAFs) for prostate cancer in MENA men ≥ 50 years.

Behavioral Risk Factor	PAF (%)
Tobacco smoking (ever vs. never)	9.5
Obesity (BMI ≥ 30 vs. <25 kg/m^2^)	3.5
Physical inactivity (lowest vs. highest activity)	6.7
High dairy product intake (highest vs. lowest)	3.1
High calcium intake (≥1000 mg/day vs. <500 mg/day)	5.6
Heavy alcohol consumption (high vs. none)	0.9
Environmental Contaminant	
Nitrate (drinking water)	17.4
THMs	5.0
Arsenic	3.1
Lead	0.7
Selenium (protective)	−4.1
PM_2.5_ (ambient air)	3.5
NO_2_ (ambient air)	2.3
Diesel exhaust (25th → 75th percentile)	0.5
Diesel exhaust (75th → 95th percentile)	0.5

**Table 4:** Cohort-based population-attributable fractions for prostate cancer in MENA men ≥50 years.

Behavioral Risk Factor	PAF (%)
Tobacco smoking (ever vs. never)	9.5
Obesity (BMI ≥30 vs. <25 kg/m^2^)	3.5
Physical inactivity (lowest vs. highest)	6.7
High dairy intake (highest vs. lowest)	3.0
High calcium intake (≥1000 vs. <500 mg/day)	5.6
Heavy alcohol consumption (high vs. none)	0.9
Environmental Contaminant	
Nitrate (drinking water)	17.4
THMs	5.0
Arsenic	3.1
Lead	0.7
Selenium (protective)	–4.1
PM_2.5_ (ambient air)	3.5
NO_2_ (ambient air)	2.4
Diesel exhaust (25th–75th percentile)	0.5
Diesel exhaust (75th–95th percentile)	0.5

## Data Availability

The data presented in this study are available on request from the corresponding author.

## Supplementary Materials

The following supporting information can be downloaded at: https://www.mdpi.com/article/10.3390/toxics14010096/s1. Table S1: Monte Carlo sensitivity analysis (mean PAF ± 95% Uncertainty Interval); Table S2: One-way sensitivity analysis (PAF ranges by RR and prevalence).

## Abbreviations

The following abbreviations are used in this manuscript:

FAO	Food and Agriculture Organization
GBD	Global Burden of Disease
MENA	Middle East and North Africa
PAF	Population attributable fraction
P0	Baseline prevalence
P1	Target prevalence
Pe	Prevalence of exposure
PIF	Potential impact fraction
PSA	Prostate-specific antigen
RR	Relative risk
THM	Trihalomethane
WHO	World Health Organization
