# Supplementary material for: Prostate Cancer in the MENA Region: Attributable Burden of Behavioral and Environmental Exposures
Source: Toxics. 2026 Jan 21;14(1):96. doi: 10.3390/toxics14010096 (PMC12845826; doi:10.3390/toxics14010096)
Supplement: Supplementary file 1 [file toxics-14-00096-s001.zip › toxics-4078890-supplementary.pdf]

Supplementary Material

Table S1. Monte Carlo Sensitivity Analysis (Mean PAF ± 95 % Uncertainty Interval)

Risk Factor / Contaminant	Mean PAF (%)	95% Uncertainty Interval (%)
**Tobacco smoking**	9.6	5.4 – 14.1
**Obesity**	3.6	–0.9 – 8.5
**Physical inactivity**	7.0	0.9 – 13.7
**High dairy intake**	3.1	–2.4 – 8.6
**High calcium intake**	5.6	0.2 – 11.1
**Heavy alcohol consumption**	0.9	0.1 – 2.0
**Nitrate**	17.4	11.9 – 22.8
**Trihalomethanes (THMs)**	4.8	–0.7 – 10.5
**Arsenic**	3.1	0.3 – 6.2
**Lead**	0.9	–2.0 – 4.0

Table S2. One-Way Sensitivity Analysis (PAF Ranges by RR and Prevalence)

Risk Factor	PAF RR Lower (%)	PAF RR Upper (%)	PAF Prev Lower (%)	PAF Prev Upper (%)
**Tobacco smoking**	4.9	13.7	7.7	11.2
**Obesity (BMI ≥ 30 vs < 25)**	–1.5	8.0	2.9	4.0
**Physical inactivity**	0.1	12.5	5.9	7.5
**High dairy intake**	–2.6	8.1	2.6	3.5
**High calcium intake**	–0.2	10.8	4.9	6.4
**Heavy alcohol consumption**	0.0	1.8	0.0	1.8
**Nitrate (drinking water)**	11.8	22.3	14.9	19.7
**Trihalomethanes (THMs)**	–0.8	10.1	4.3	5.7
**Arsenic**	0.1	6.0	2.3	4.0
**Lead**	–2.1	3.4	0.5	0.9
